# ZebraMap: A Multimodal Rare Disease Knowledge Map with Automated Data Aggregation & LLM-Enriched Information Extraction Pipeline

**DOI:** 10.3390/diagnostics16010107

**Published:** 2025-12-29

**Authors:** Md. Sanzidul Islam, Amani Jamal, Ali Alkhathlan

**Affiliations:** 1 Department of Computer Science, FCIT, King Abdulaziz University, Jeddah 21589, Saudi Arabia; atjamal@kau.edu.sa (A.J.); analkhathlan@kau.edu.sa (A.A.); 2 Department of Computer Science, FSIT, Daffodil International University, Birulia 1216, Bangladesh; 3 Center of Research Excellence in Artificial Intelligence and Data Science, King Abdulaziz University, Jeddah 21589, Saudi Arabia

**Keywords:** multimodal dataset, rare disease diagnosis, knowledge map, case reports, LLM applications, retrieval-augmented generation (RAG), clinical images, structured clinical records

## Abstract

**Background:** Rare diseases often lead to delayed diagnosis because clinical knowledge is fragmented across unstructured research, individual case reports, and heterogeneous data formats. This study presents ZebraMap, a multimodal knowledge map created to consolidate rare disease information and transform narrative case evidence into structured, machine-readable data. **Methods:** Using Orphanet as the disease registry, we identified 1727 rare diseases and linked them to PubMed case reports. We retrieved 36,131 full-text case report articles that met predefined inclusion criteria and extracted publication metadata, patient demographics, clinical narratives (cases), and associated images. A central methodological contribution is an automated large language model (LLM) structuring pipeline, in which free-text case reports are parsed into standardized fields, such as symptoms, diagnostic methods, differential diagnoses, treatments, and outcome that produce structured case representations and image metadata matching the schema demonstrated in our extended dataset. In parallel, a retrieval-augmented generation (RAG) component generates concise summaries of epidemiology, etiology, clinical symptoms, and diagnostic techniques by retrieving peer-reviewed research to enhance missing disease-level descriptions. **Results:** The final dataset contains 69,146 structured patient-level case texts and 98,038 clinical images, each linked to a particular patient ID, disease entry, and publication. Overall cosine similarity between curated and generated text is 94.5% and performance in information extraction and structured data generation is satisfactory. **Conclusions:** ZebraMap provides the largest openly accessible multimodal resource for rare diseases and enables data-driven research by converting narrative evidence into computable knowledge.

## 1. Introduction

Rare diseases, as defined in the European Union, are those affecting fewer than 1 in 2000 individuals [1]; and rare diseases, though individually uncommon, collectively affect an estimated 3.5–5.9% of the global population, amounting to 263–446 million people worldwide at any given time [2,3]. According to a comprehensive analysis of the Orphanet database, there are over 6000 distinct rare diseases, with approximately 72% being of genetic origin and 70% manifesting in childhood [1]. These conditions are individually infrequent but, in aggregate, present a substantial global health burden, contributing to significant morbidity, mortality, and unmet medical needs. Misdiagnosis and diagnostic delays are common challenges for people with rare diseases. Studies show that misdiagnosis rates can be very high: in China, nearly 73% of adult rare disease patients were misdiagnosed before receiving a definitive diagnosis [4,5], while in an Australian study, 27% of children with rare diseases initially received a wrong diagnosis [6]. In Europe, misdiagnosis is a significant factor contributing to diagnostic delays, with over half of patients experiencing a delay in diagnosis [7].

The average time to receive a correct diagnosis for a rare disease typically ranges from 4.3 to 6.2 years, depending on the country and population studied [7]. For example, the average time to diagnosis in Europe is about 4.7 years [7], in Spain it is 6.2 years [8], and in China it is 4.3 years [4]. These prolonged diagnostic journeys often involve consulting multiple healthcare professionals and can lead to significant emotional, social, and health consequences for patients and families [6,7,8]. Despite recent advances in genomics and data-driven approaches, less than 6% of rare diseases currently have an approved treatment [9], underscoring the persistent gap in clinical management and therapeutic development. These challenges highlight the pressing need for integrated resources and innovative approaches that can improve diagnostic accuracy and accelerate research in the rare disease field.

### 1.1. Background

Rare diseases, though individually uncommon, collectively affect hundreds of millions of people worldwide and present a formidable challenge to healthcare systems due to their clinical heterogeneity, low prevalence, and the limited awareness among clinicians [6,7,10,11,12]. The delayed journey to a correct diagnosis, often termed the diagnostic odyssey, can span years, with patients frequently experiencing misdiagnoses, repeated consultations, and significant psychosocial and economic burdens [6,8,10,13,14,15]. This prolonged diagnostic process is exacerbated by the rarity of individual conditions, the diversity of clinical presentations, and the fragmentation of biomedical knowledge across disparate sources [7,10,16,17,18,19].

Traditional diagnostic approaches for rare diseases have relied heavily on clinical expertise, heuristic reasoning, and the aggregation of case reports and research, but these methods are often insufficient given the vast number of rare diseases and the limited exposure most clinicians have to them [6,10,16,17,20,21,22,23]. Recent advances in genomics, generative intelligence, and computational methods including artificial intelligence (AI), machine learning, and knowledge map technologies have begun to transform the landscape of rare disease diagnosis and research [21,22,24,25,26,27,28]. These innovations enable the integration of heterogeneous data types, from disease general information and clinical phenotypes to real-world case evidence and imaging, offering new opportunities for earlier and more accurate diagnoses.

Despite these advances, significant gaps remain. Many rare diseases still lack comprehensive molecular characterization, and a substantial proportion of patients remain undiagnosed even after extensive genetic testing [21,22,24,28,29]. The need for resources that can systematically combine structured biomedical knowledge with real-world case data is increasingly recognized as essential for both clinical decision support and research [10,19,26]. Initiatives such as Orphanet and the development of multimodal knowledge maps represent important steps toward addressing these challenges by linking diseases, phenotypes, cases, and evidence in a unified, computable framework. Still, controversy remains over how best to integrate multimodal evidence, how to fill gaps in disease-level information, and how to ensure reproducibility and provenance when applying generative AI in biomedical contexts. In this context, we developed ZebraMap, a multimodal knowledge map integrating Orphanet-based disease definitions, PubMed-linked case reports, patient demographics, clinical images, structuring case information, and retrieval-augmented generation (RAG) completions of missing fields. This resource demonstrates the feasibility of large-scale multimodal integration and establishes ZebraMap as a foundation for reducing diagnostic delays and improving rare disease research.

### 1.2. Research Importance

Aggregating and structuring rare disease data, especially using multimodal approaches that combine clinical, genomic, and other data types, is critically important for advancing research, diagnosis, and therapy development. Integrative platforms and knowledge maps enable researchers to identify research gaps, analyze funding patterns, and generate scientific evidence more efficiently, accelerating the pace of rare disease discovery and therapeutic innovation [30,31]. Multimodal data aggregation supports the creation of harmonized, interoperable databases, which are essential for understanding disease natural history, improving diagnostic accuracy, and facilitating large-scale collaborative studies, as seen in initiatives like RD-Connect and Solve-RD [31,32,33].

Standardized and accessible data infrastructures also make it possible to pool patient cohorts, support real-world evidence generation, and inform evidence-based personalized medicine, which is especially valuable given the small and dispersed nature of rare disease populations [34,35]. Synthetic data generation and advanced analytics further enhance the ability to train AI models, simulate clinical trials, and ensure privacy while expanding research opportunities [32]. Overall, well-structured, multimodal data aggregation is foundational for overcoming the challenges of rare disease research and for driving progress in diagnosis, treatment, and policy development [30,31,36].

### 1.3. Aim & Objectives

- **Aim 1**: Construct a reproducible and scalable pipeline to build a comprehensive rare disease knowledge map by integrating Orphanet, PubMed, case reports, and clinical images.
- **Aim 2**: Rigorously evaluate LLM-RAG models for structuring, synthesizing, and imputing missing or incomplete fields in rare disease data, directly benchmarking the effectiveness of generative AI in addressing knowledge gaps across heterogeneous biomedical sources.
- **Aim 3**: Design and implement baseline evaluation tasks and protocols to systematically reveal both the strengths and limitations of LLM-RAG approaches in the context of multimodal rare disease knowledge integration, data curation, and diagnostic support.

### 1.4. Research Contributions

This research develops a reproducible methodology for constructing an enriched, multi-modal knowledge base for rare diseases by integrating heterogeneous sources (Orphanet, PubMed, clinical case reports, and medical images). Beyond collection, we design AI-assisted curation strategies (including retrieval-augmented generation and ontology alignment) to fill missing information such as symptoms and etiology while ensuring provenance and interpretability. Also, an automated workflow of information extraction and classification of case reports and images is developed to provide more structured insights of disease cases. This resource not only advances the availability of high-quality rare disease data but also enables novel evaluation tasks and explainable LLM approaches for rare disease diagnosis, establishing both a dataset and a methodological framework as contributions.

- Introduced ZebraMap, a large-scale multimodal knowledge map for rare diseases encompassing 1727 diseases, 36,131 case reports, and 98,038 clinical images.
- Developed an automated pipeline for extracting and classifying information from case reports and medical images to enhance disease-level insights.
- Leveraged retrieval-augmented generation (RAG) from the linked literature to fill gaps in disease-level data, ensuring both reliability and provenance.
- Established baseline evaluation procedures for rare disease data aggregation, highlighting both the strengths and remaining challenges of multimodal and generative approaches.

## 2. Literature Review

The development of multimodal biomedical knowledge maps has emerged as a transformative approach for integrating heterogeneous data such as text, case reports, and medical images to advance rare disease research and diagnostic support. Rare diseases, affecting fewer than 1 in 2000 individuals, present unique challenges due to their heterogeneity, limited clinical expertise, and data scarcity, often resulting in delayed or inaccurate diagnoses [37,38,39]. Foundational knowledge bases like Orphanet, PubMed, and the Human Phenotype Ontology (HPO) have provided structured resources, but they face limitations in coverage, interoperability, and real-world applicability [40]. Recent advances in multimodal data fusion, deep learning, and large language models (LLMs), including retrieval-augmented generation (RAG), have enabled more robust knowledge extraction, graph completion, and clinical decision support [41,42,43,44]. However, the integration of diverse data modalities, especially case reports and medical images, introduces new challenges related to data quality, bias, privacy, and reproducibility [27,45,46]. This review synthesizes evidence from high-impact studies, systematic reviews, and evaluate datasets to critically assess the state of the art, highlight consensus and controversies, and identify research gaps in the field of multimodal biomedical data for rare diseases.

A comprehensive search was conducted encompassing Semantic Scholar, PubMed, and additional biomedical databases. The search strategy targeted peer-reviewed studies, systematic reviews, and datasets addressing rare disease knowledge bases, multimodal knowledge map construction, LLM-based curation, diagnostic evaluation, the role of case reports, and ethical considerations. In total, 1037 papers were identified, 413 were screened, 258 were deemed eligible, and the top 50 most relevant papers were included in this review.

Table 1 summarizes the stages and record counts of the literature search and selection process described above.

Nine distinct search strategies were implemented to comprehensively address foundational knowledge bases, multimodal knowledge construction, LLM and RAG applications, datasets and benchmarks, case reports, challenges, ethical considerations, and research gaps.

### 2.1. Existing Rare Disease Knowledge Bases and Their Limitations

Foundational resources such as Orphanet, MONDO, and HPO are widely used for disease research, providing structured phenotype, genotype, and disease entity data [10,40]. However, these knowledge bases face limitations in terms of incomplete coverage, inconsistent data standards, and challenges in integrating real-world clinical data and imaging [10,40,47], especially for rare diseases. Disease registries and electronic health records (EHRs) offer additional data but are often siloed, lack interoperability, and present privacy concerns [47,48].

Table 2 summarizes major rare disease data repositories, outlining their content, primary strengths, and key limitations regarding structured, multimodal, and case-level data integration. This comparison highlights gaps in existing resources, including limited linkage to medical images, case reports, and real-world clinical narratives that motivate further development of comprehensive, integrative rare disease knowledge platforms.

### 2.2. Datasets and Benchmarks for Rare Disease Diagnosis

Publicly available datasets and benchmarks for rare disease diagnosis remain limited, especially for multimodal approaches [44,45,50,51,52]. Most studies rely on private or disease-specific datasets, with a predominance of image and omics data [46,51,52]. Multimodal models consistently outperform unimodal models in diagnostic accuracy, but lack of standardized benchmarks and external validation hinders reproducibility and generalizability [27,51,52].

Table 3 highlights that existing public rare disease datasets are typically focused on disease- or modality-specific data, and very few offer comprehensive, case-linked datasets containing structured, imaging, and narrative modalities together. Most resources either lack clinical detail at the case level or do not integrate imaging with text and structured phenotype data. In contrast, our work addresses this gap by providing a multimodal, case-based dataset linking narrative, image, and structured information, thereby enabling more holistic research and benchmarking in rare disease diagnosis.

### 2.3. Methodologies for Knowledge Mapping & Rare Disease Research

Recent advances in multimodal data fusion and deep learning have enabled the integration of text, case reports, and medical images into unified knowledge bases, delivering substantial improvements in diagnostic and classification accuracy across biomedical tasks. For example, multimodal fusion methods, particularly intermediate (joint) fusion, consistently outperform unimodal approaches, with reported increases in diagnostic accuracy ranging from 5–10% in benchmark evaluations [14,41,44,57]. Intermediate fusion strategies are notably effective at capturing complex interactions between heterogeneous data types compared to early or late fusion [14,41,42,58]. Graph neural networks (GNNs) and transformer-based architectures, such as BioBERT and related models, are becoming increasingly central for extracting and reasoning over multimodal biomedical knowledge, driving significant advances in rare disease research and phenotype-genotype association tasks [42,44,59,60]. Nevertheless, challenges remain, including accurate alignment of data modalities, imputation of missing or incomplete data, and maintaining scalability as data volume increases [41,42,43,60].

Large language models (LLMs) and retrieval-augmented generation (RAG) approaches are increasingly used for tasks such as automated knowledge base completion, information extraction from unstructured case reports and the literature, and curation of rare disease knowledge resources [38,39,43,61,62]. These methods have demonstrated gains in extraction accuracy and knowledge synthesis, often facilitating precision medicine and explainable decision support with accuracies exceeding 80% in some settings [39,43,61]. However, some generated knowledge remains susceptible to hallucinations, inconsistencies, and bias, especially when source data is limited or heterogeneous, and thorough external validation is often lacking [38,39,61,62]. Ongoing research highlights the need for standard evaluation and systematic cross-validation to ensure reliability and clinical utility of these AI-driven methodologies.

### 2.4. Challenges, Limitations, and Ethical Considerations

Case reports are a valuable source of rare disease knowledge, capturing unique phenotypes and diagnostic journeys [37,38,39]. Systematic mining of case reports using NLP and LLMs has enabled the extraction of structured knowledge for knowledge map construction [38,39,40]. However, case reports are subject to publication bias, lack of standardization, and limited generalizability [38,39,40,46].

Key challenges include data privacy, licensing, bias, and reproducibility. Multimodal datasets often lack standardized consent and data-sharing agreements, and there are concerns about algorithmic bias, especially for underrepresented populations. Reproducibility is hampered by proprietary datasets, lack of open evaluation, and insufficient reporting of methods [27,45,46]. Table 4 below summarizes key studies on multimodal knowledge maps in rare disease diagnosis, highlighting challenges.

Table 5 summarizes key claims in the literature regarding multimodal knowledge maps and rare disease diagnosis. Multimodal approaches consistently outperform unimodal ones in predictive accuracy. However, challenges persist, including limited data availability, lack of reproducible benchmarks, and issues with LLMs such as hallucination and bias. These points highlight both the progress and ongoing obstacles in the field.

Multimodal biomedical knowledge maps represent a powerful paradigm for rare disease research and diagnostic support, enabling the integration of heterogeneous data and advanced AI methods. While significant progress has been made, challenges remain in data standardization, external validation, ethical data sharing, and explainability. Addressing these gaps will be critical for realizing the full potential of knowledge bases in rare disease diagnosis and care.

## 3. ZebraMap Dataset: Structure and Extensions

**Figure 1:**
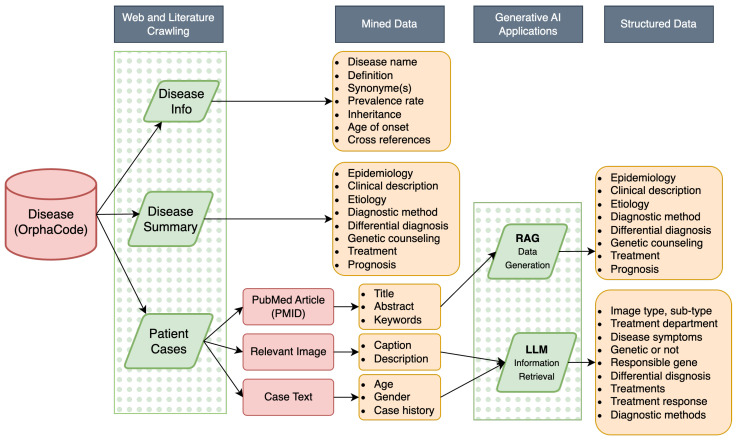
Knowledge Map Relations Overview.

ZebraMap is a multimodal knowledge base purpose-built for rare disease research, supporting both comprehensive disease profiling and granular exploration of patient cases from the biomedical literature. At its core, ZebraMap is organized as a deeply structured JSON knowledge map, where each disease entity (keyed by OrphaCode) links to machine-readable disease metadata, extracted and structured clinical case reports, and associated medical images. The schema integrates curated, extracted, and generative content, balancing interpretability with completeness (see Figure 1 for a logical overview).

### 3.1. Knowledge Base Backbone: Disease–Cases–Images Mapping

The core structure of the ZebraMap dataset is organized around four key files, complemented by an images directory:

- ZebraMap.json: This file serves as the main knowledge base, where each top-level key is a disease. For each disease, the record contains:
  – AboutDisease: Disease-centric information fields, including originally curated metadata (such as Name, Definition, Synonyms, Prevalence, Inheritance, Onset, and cross-references to ontological resources).
  – Cases: A dictionary or list linking to disease-associated case reports. Each case is identified by a unique CaseID, and contains:
    * Free-text narrative or structured case summary.
    * Case metadata, such as Age, Sex, genetic findings, diagnosis, clinical intervention, and outcome (where available).
    * Reference(s) to the supporting literature via linkage to the primary source (e.g., PMID, PMCID).
    * Optionally, references to associated image files (by image ID or file name) with associated caption and description.
- For disease records in augmented_disease_data.json, missing or incomplete fields are filled using retrieval-augmented generation (RAG) with advanced language models, leveraging the relevant literature as context. Generated content is marked with a G_ prefix (e.g., G_Epidemiology) and includes explicit provenance, typically referencing the PMCID/PMID of the source literature.
- The extended file of extracted cases, structured with LLM pipeline is stored in structured_cases.json. Each entry contains detailed, normalized fields: patient demographics, clinical presentation, diagnosis, outcomes, and links to disease (by OrphaCode/key), case (by CaseID), and source article. This file enables efficient search, filtering, and statistical analysis of the underlying case population.
- literature_metadata.json: A comprehensive metadata file containing bibliographic details for each research source referenced in cases. Each article entry is identified by a unique article key (e.g., PMID or PMCID), and includes title, authors, year, journal, DOI, and article type, as well as the license information.

The images referenced in cases are linked to their corresponding digital files in the images/directory, with metadata (caption, source, figure label, etc.) supporting direct traceability from disease → case → as follows: → image.

This data schema ensures both high-level and granular analysis: all disease-centric information and their connections to evidence (cases, figures, research) are available in a machine-readable, explicitly cross-referenced structure. The separation between knowledge base (ZebraMap.json), structured case entries (structured_cases.json), filled disease data (augmented_disease_data.json), and literature metadata (literature_metadata.json) facilitates flexible querying, data integration, and development of informatics or AI applications. The modular organization also supports automation, reproducibility, and transparent provenance tracking across the entire dataset.

### 3.2. Exploratory Data Statistics

Table 6 presents an overview of the main characteristics of the **ZebraMap** dataset as structured across its four foundational files. The central knowledge base, ZebraMap.json, comprises 1727 rare diseases, with each entry linking directly to disease-level metadata, curated and generated case text, lists of associated cases and medical images. structured_cases.json catalogs 69,146 individual clinical case reports, each normalized to a common schema and mapped to its corresponding disease and literature source for comprehensive analysis. The literature_metadata.json file integrates more than 36,131 unique literature articles, providing complete bibliographic metadata for source traceability and dataset integrity. The augmented_disease_data.json file provide extended version of disease information, filled with augmented data generation. Out of the total patient cases, 45,198 are directly associated with one or more images, and the complete images repository encompasses 98,038 image files, each referenced both from the case and literature metadata. This structure enables granular, cross-referenced exploration of rare diseases, cases, literature, and images, facilitating advanced queries, reproducibility, and downstream development of AI and informatics tools.

## 4. Materials and Methods

**Figure 2:**
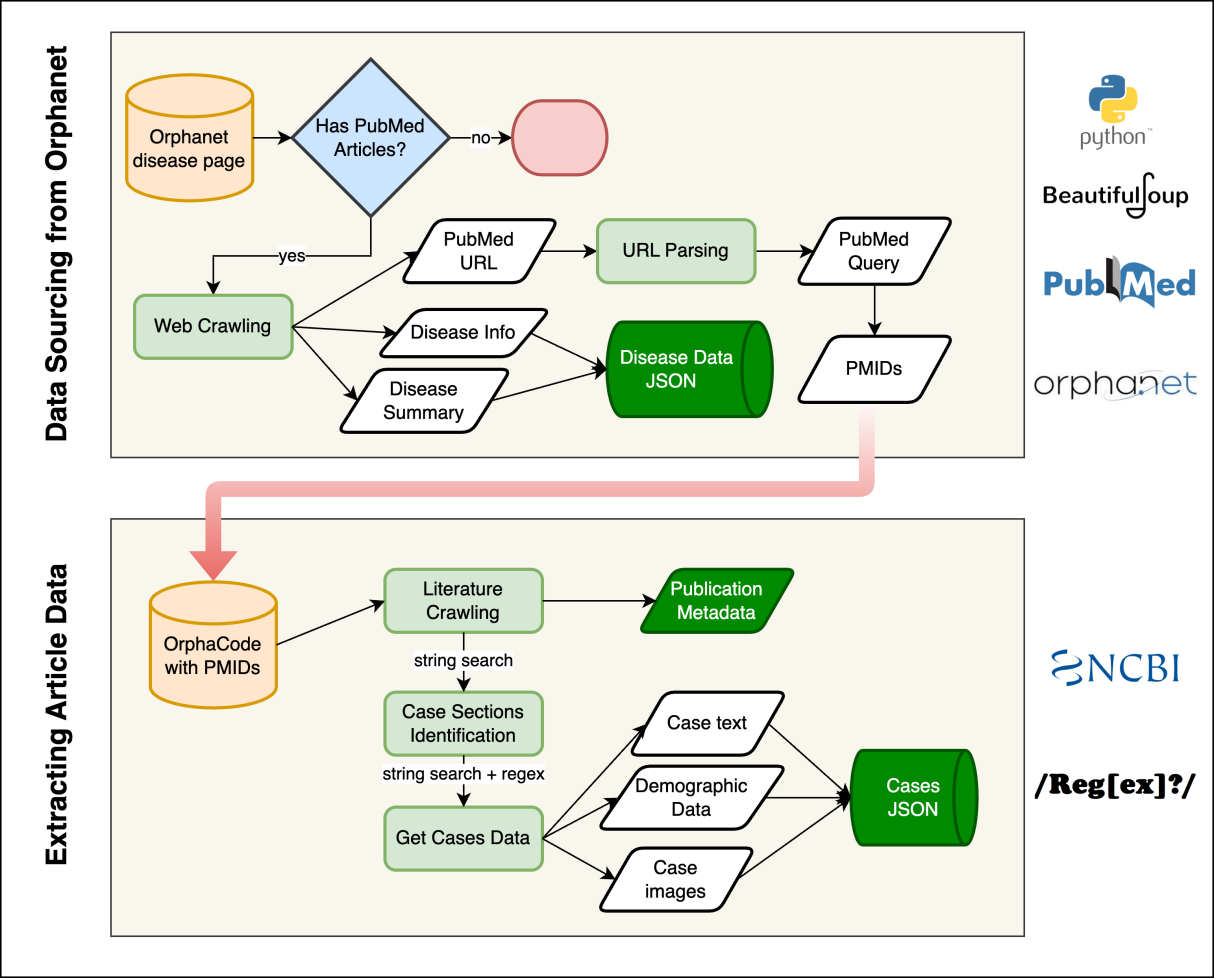
Web & Literature Crawling Methodology.

**Figure 3:**
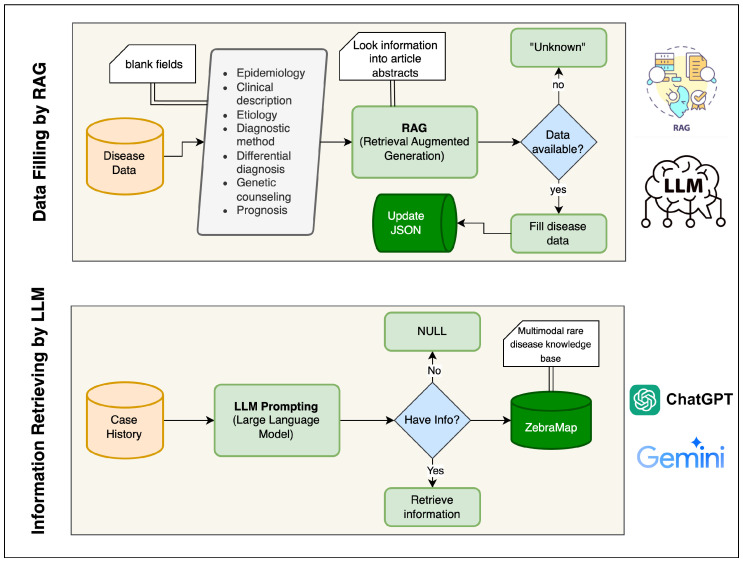
Generative AI Applications Overview.

The development of the **ZebraMap** dataset followed a hybrid pipeline that combines deterministic web and literature crawling with probabilistic generative augmentation. The overall architecture of the workflow is illustrated in Figure 2 and Figure 3.

The pipeline consists of five sequential modules: (1) Orphanet indexing and disease metadata retrieval; (2) PMID harvesting; (3) full-text PDF/PMC retrieval; (4) deterministic extraction of cases, text, and figures; (5) LLM- and RAG-based structuring and augmentation; and (6) final integration into ZebraMap. Each module passes standardized objects to the next stage, ensuring continuity and traceability.

All modules were implemented in Python 3.11, with each stage encapsulated as an independent script to ensure modularity, reproducibility, and transparent provenance tracking.

### 4.1. Orphanet → PMID Retrieval

The initial step of the pipeline involves the automated extraction of disease data from the Orphanet repository. Each disease record is uniquely indexed by its *OrphaCode*, forming the foundational disease set:

(1) $$D=\{d_{1},d_{2},\ldots ,d_{N}\},\;N\approx 2000.$$

For each disease $d_{i}$, we collect two groups of structured metadata:

$$I_{i}=\left\{\mathrm{Name},\mathrm{Definition},\mathrm{Synonyms},\mathrm{Prevalence},\mathrm{Inheritance},\mathrm{Onset},\mathrm{CrossRefs}\right\},$$

$$\begin{array}{c} S_{i}=\{\mathrm{Epidemiology},\;\mathrm{ClinicalDescription},\;\mathrm{Etiology},\;\mathrm{DiagnosisMethods},\\ \;\mathrm{DifferentialDiagnosis},\;\mathrm{Counseling},\;\mathrm{Treatment},\;\mathrm{Prognosis}\}. \end{array}$$

All fields are parsed using BeautifulSoup4, with normalization performed via regular expressions. Polite crawling routines, including delay and retry mechanisms, ensure compliance with Orphanet terms and reproducible checkpoints.

The extraction results in hierarchical JSON objects of the following form:

(2) $${\mathrm{DiseaseData}}_{i}=\left(I_{i},S_{i},{\mathrm{PMIDs}}_{i}\right),$$

stored persistently for downstream modules. At this stage, we establish the set of candidate diseases and their core metadata, which serve as the anchor for subsequent literature retrieval and integration processes.

To ensure the dataset’s quality and traceability, we applied rigorous filtering criteria to determine eligible case report articles. Starting from each disease’s “Publications in PubMed” links in Orphanet, we included only articles that were: (1) available as **free full text**; (2) labeled as “Case Reports”; (3) in **English**; and (4) focused on **human** subjects. Multilingual or non-standard formats (such as abstracts without full text, image-only content, or unparseable scans) were automatically excluded. Articles with missing critical metadata (e.g., publication year, PMID/PMCID, or author list) were filtered out to maintain provenance. Duplicate entries were detected by cross-referencing PMIDs, PMCIDs, and DOIs, ensuring each case report is uniquely represented. This systematic filtering maximized dataset integrity and reproducibility.

The overall Orphanet-to-PMID linking is summarized in Figure 2 (upper panel).

### 4.2. PMID → PMC Full-Text Acquisition

Building upon the disease records extracted in the previous step, we next retrieve evidence and literature associations. When Orphanet entries contain references to PubMed, their corresponding PMIDs are parsed using URL pattern recognition and NCBI E-utilities:

(3) $${\mathrm{PMIDs}}_{i}=f_{\mathrm{Entrez}}\left(\mathrm{Name}(d_{i})\right)$$

where $f_{\mathrm{Entrez}}$ refers to an NCBI search, typically limited to titles and abstracts for specificity. This step directly links each disease to a curated set of PubMed articles, establishing the literature basis required for subsequent data enrichment and full-text acquisition.

The resulting PMIDs from Orphanet are not only embedded within the disease-level JSON object, but are also queued for full-text processing in the next stage of the pipeline. This ensures tight continuity between disease indexing and literature sourcing.

### 4.3. Publication Metadata (XML) Parsing

With the curated list of PMIDs established for each disease, the pipeline proceeds to enrich each article by acquiring comprehensive publication metadata. For every PMID associated with a disease, we collect metadata through NCBI E-utilities and targeted HTML scraping:

(4) $$M_{ij}=\left\{\mathrm{PMCID},\mathrm{DOI},\mathrm{Title},\mathrm{Abstract},\mathrm{Year},\mathrm{MeSH},\mathrm{Keywords}\right\},$$

where *i* is the disease index and *j* the sequential article instance.

For each disease, all such metadata are aggregated as follows:

(5) $$M_{i}=\overset{k_{i}}{\underset{j=1}{⋃}}M_{ij}.$$

These metadata files maintain explicit mapping among diseases, PubMed/PMC articles, and their respective attributes, and are serialized per disease as standalone JSON records.

This seamless linking—from Orphanet disease records, to curated PMIDs, to enriched article metadata—forms the backbone of traceability in the pipeline, ensuring that subsequent downstream processing (e.g., clinical case extraction, RAG-based augmentation) can reference both provenance and supporting evidence at every step.

### 4.4. Case Report Extraction from PMC Full-Texts

To systematically harvest patient-level data, we process the full-text XMLs of PubMed Central (PMC) articles associated with each disease. The primary objective at this stage is to extract individual clinical case reports, along with their associated demographic features and references to figures. Given the great heterogeneity of article formats and conventions, dedicated algorithms identify and segment out clinical cases.

#### 4.4.1. Section Detection and Case Identification

Our parser first locates candidate case sections by searching for section headings and subheadings containing indicative keywords such as “Case”, “Patient”, “Observation”, or “Clinical report”. Both exact and fuzzy matches are used, leveraging regular expressions and section node analysis to accommodate format variability across journals and years. Once candidate sections are isolated, additional logic splits multi-case reports, for example by numerically labeled subheadings or repeated introduction patterns (e.g., “Case 1”, “Patient 2”). This approach allows for reliable identification of each discrete patient narrative within this article.

Each extracted case is indexed for traceability with a composite identifier:

(6) $${\mathrm{CaseID}}_{ijc}={\mathrm{PMCID}}_{ij}\text{\_}c,$$

where ${\mathrm{PMCID}}_{ij}$ denotes the *j*th article for disease *i*, and *c* is the sequential number of the case within this article.

#### 4.4.2. Feature Extraction and Normalization

For each identified case, the entire narrative text is retained (CaseText), and regular-expression-based extraction procedures are used to detect commonly reported demographic attributes such as age and gender. Attempts are made to standardize reported ages (e.g., “6-year-old male”), infer gender pronouns or lexemes, and associate all image or figure number references contained within each narrative.

Each case data object is thus represented as follows:

(7) $$C_{ijc}=\left\{\;\mathrm{CaseText},\;\mathrm{Age},\;\mathrm{Gender},\;\mathrm{ImageRefs}\;\right\}.$$

These objects are stored per disease and per article for downstream analysis and integration.

### 4.5. Extraction and Mapping of Clinical Images

A complementary effort targets the retrieval of clinical images (e.g., photographs, radiographs, histology) and precise mapping of figures to cases. Image metadata are extracted from the structured XML nodes, focusing on <fig> elements, their associated <caption>s, and file references (e.g., image href or graphic tags). Figure captions are parsed to determine their type and to extract references mentioning particular case numbers, anatomical sites, or illustrative events.

#### 4.5.1. Linking Images to Case Narratives

To unambiguously connect images to extracted case reports, captions and in-text figure citations are scanned for explicit references (such as “see Figure 2, Patient 1”). The parser cross-references figure numbers in both the case narratives (via the ImageRefs collected above) and the fig metadata, assigning image IDs to one or more specific cases wherever a match is found.

#### 4.5.2. Image Downloading and Organization

For each matched image, the corresponding graphic file is programmatically retrieved and downloaded from PMC servers. Images are organized deterministically in a nested folder hierarchy: /images/{DiseaseID}/{PMCID}/, supporting efficient mapping between images, diseases, and articles.

The outlined workflow ensures that clinical cases and their visual evidence are tightly linked and accessible for further processing and semantic analysis. The lower panel of Figure 2 summarizes the main steps in this extraction process.

### 4.6. RAG-Based Missing Disease Section Completion

Many Orphanet disease summaries contain incomplete textual segments. To address this, missing fields are inferred using a Retrieval-Augmented Generation (RAG) framework. For each missing attribute $f_{m}\in S_{i}$, a set of relevant abstracts $\left\{A_{i1},\ldots ,A_{ik}\right\}$ is retrieved and passed as evidence to a Gemini-based language model:

(8) $${\hat{s}}_{if_{m}}=\mathrm{LLM}\left(f_{m},\left\{A_{i1},A_{i2},\ldots ,A_{ik}\right\}\right).$$

The final disease summary is updated as follows:

(9) $$S_{i}^{\prime }=S_{i}\cup \left\{\left(f_{m},{\hat{s}}_{if_{m}}\right)\right\},$$

producing the output file in another JSON file. Each generated text segment is annotated with its provenance metadata (source=RAG, model=gemini-2.5-flash-lite). The upper block of Figure 3 presents this workflow. The AI prompt used for the RAG is given in Supplementary Material.

### 4.7. LLM-Based Structuring of Patient Cases

A single-pass large language model (LLM) pipeline was developed to convert unstructured clinical case narratives into standardized, machine-readable records. For each case, the system aggregates all available contextual inputs, including the free-text narrative, pre-extracted demographics, disease name, and image metadata into a unified prompt. This avoids multi-stage inference drift and ensures consistent cross-field extraction.

#### 4.7.1. Prompt Construction

Each prompt contains: (i) the case narrative; (ii) provided age and gender (used as authoritative overrides); (iii) the Orphanet-linked disease name; and (iv) identifiers and descriptive metadata for associated images. The gemini-2.5-flash-lite model is instructed to return a strictly defined JSON structure. Medical departments must be chosen from a fixed set, and image categories must map to predefined imaging domains and subtypes. The model is instructed to avoid unsupported inferences and to leave fields empty when evidence is absent. The prompt is given in Supplementary Material.

#### 4.7.2. One-Shot Extraction

The model extracts four main components from each case: medical department, demographics, diagnostic information, and image classification.

(1) Medical Department.

One department is selected from a controlled list to ensure terminological consistency. The available department options are: Neurology, Pediatrics, Gastroenterology, General Medicine, Cardiology, Dermatology, Oncology, Orthopedics, Pulmonology, Endocrinology, Psychiatry, Nephrology, Rheumatology, Infectious Disease, Obstetrics and Gynecology, Urology, Ophthalmology, and Otolaryngology.

(2) Demographics.

Age, gender, congenital onset, genetic status, and putative genes are extracted when present. Approximate or non-integer ages are normalized by

$$\hat{a}=\max\left(1,\left\lfloor a\right\rfloor \right).$$

Missing demographics default to $\hat{a}=1$ and “Unknown” gender.

(3) Diagnostic Information.

The output includes symptoms, diagnostic procedures, treatments, treatment response (*Yes*, *No*, *Partial*, *Unknown*), and explicitly stated differential diagnoses.

(4) Image Classification.

For each image, the model returns {FileID, ImageType, ImageSubType}. Unassigned images are appended with empty type fields. All entries are merged with the original metadata to preserve file paths and identifiers.

In summary, ZebraMap unifies multimodal knowledge—from real patient narratives to richly annotated clinical images and supporting metadata—into a single, rigorously standardized resource. The dataset’s comprehensiveness is reinforced by systematic validation protocols at each stage of data acquisition and curation. Advanced language models are employed not only to extract missing details but also to transform unstructured case material into structured, machine-actionable records.

Figure 2 and Figure 3 provide an overview of this pipeline: Figure 2 outlines the rule-based collection and document-linking stages, while Figure 3 highlights the generative and structuring procedures. Through the integration of deterministic data mining and LLM-driven augmentation, ZebraMap achieves broad coverage and semantic precision, establishing a unique resource for the study of rare diseases.

## 5. Data Validation

The data validation process in our study was designed to reflect the two principal components of ZebraMap: disease knowledge summaries and structured case-level data. For disease summaries, all data sources used in the Retrieval-Augmented Generation (RAG) pipeline were strictly limited to peer-reviewed, open-access articles from PubMed. Each generated disease field is explicitly linked to its source PubMed reference, ensuring full transparency, traceability, and scientific integrity. To further validate accuracy and biomedical correctness, we systematically compared all RAG-generated narrative fields against authoritative records from the Orphanet database. These comparisons utilized both quantitative and semantic similarity measures to assess fidelity to expert-curated knowledge.

For clinical cases, we employed the Gemini 2.5 Flash Lite large language model to structure and annotate unstructured case narratives and associated image metadata. This structuring included the extraction of key clinical details, the assignment of standardized field values, and the categorization of clinical images. The following sections provide a detailed description of the specific validation methodologies and results for both the RAG-based disease knowledge construction and the LLM-based case structuring components.

Our image extraction strategy relies on automated parsing of the digital full-text articles, where figures are detected, extracted, and linked to their bibliographic context using rule-based heuristics. Supported file types include JPEG, PNG, and TIFF, which are preserved in their original format and referenced by unique IDs in the knowledge map. Anonymization is ensured by systematically excluding images containing direct patient identifiers; only images lacking personally identifying information, as determined by automated filters are included. Quality control is conducted through standardized file integrity checks and visual inspections of a random sample of extracted images. To prevent misalignment, each image is linked directly to the cited case narrative and publication metadata via persistent file and article identifiers, ensuring traceable and unambiguous mapping between text and images throughout the dataset.

### 5.1. Validation of Disease Data with RAG

To evaluate the semantic fidelity of the generated disease summaries, we conducted a systematic validation using a random sample of 100 diseases for which all curated narrative fields were available. For each selected disease, the model generated a full set of textual fields (e.g., *Definition*, *Clinical Description*, *Etiology*), which were quantitatively compared against their expert-curated counterparts.

#### 5.1.1. Text Pairing

Each disease record contained a DiseaseSummary object with both original and generated versions of each field, the latter identified by the prefix “G_”. For a field *f*, we constructed a valid comparison pair $\left(T_{f},{\hat{T}}_{f}\right)$ only when both texts were present, non-empty, and the generated text was not a placeholder value such as “Unknown”. This ensured that similarity measurements reflected genuine content comparisons.

#### 5.1.2. Embedding Representation

Both texts in each pair were transformed into dense semantic embeddings using a sentence-level embedding model specialized for the clinical domain, ClinicalBERT. Formally,

(10) $$e_{f}=E\left(T_{f}\right),\;{\hat{e}}_{f}=E\left({\hat{T}}_{f}\right)$$

where E(·) denotes the embedding function mapping a variable-length text sequence to a normalized vector in $R^{d}$. These embeddings capture semantic information enabling meaningful comparison between curated and generated descriptions.

#### 5.1.3. Similarity Measurement

Semantic similarity between the curated and generated texts was quantified using cosine similarity:

(11) $$\mathrm{Sim}\left(T_{f},{\hat{T}}_{f}\right)=\cos\left(\theta \right)=\frac{e_{f}\cdot {\hat{e}}_{f}}{∥e_{f}∥\;∥{\hat{e}}_{f}∥}$$

which measures the cosine of the angle θ between the two embedding vectors. Since the embeddings were normalized, the expression simplifies to:

(12) $$\mathrm{Sim}\left(T_{f},{\hat{T}}_{f}\right)=e_{f}\cdot {\hat{e}}_{f}.$$

The resulting similarity values lie in the interval [−1,1], with higher values indicating stronger semantic agreement.

#### 5.1.4. Field-Level Aggregation

For each field type *f*, we aggregated the similarity scores across all diseases with valid comparison pairs. The field-level mean similarity was computed as

(13) $${\bar{S}}_{f}=\frac{1}{N_{f}}\sum_{i=1}^{N_{f}}\mathrm{Sim}\left(T_{f,i},{\hat{T}}_{f,i}\right),$$

where $N_{f}$ denotes the number of diseases contributing valid text pairs for field *f*. This aggregate measure reflects the overall alignment between the generated content and the expert-curated ground truth for each narrative dimension.

The resulting field-wise similarity metrics serve as quantitative evidence of the reliability and semantic consistency of the RAG system across core disease-description fields.

### 5.2. Validation of Data Structuring & Information Retrieval with LLM

To evaluate the capability of large language models (LLMs) in clinical data structuring and information retrieval, we designed a systematic framework for extracting symptoms from unstructured biomedical case descriptions and mapping them to standardized Human Phenotype Ontology (HPO) concepts. The evaluation was conducted on a set of 100 randomly selected diseases for which complete Orphanet phenotype profiles were available.

#### 5.2.1. Data Sources

Two complementary data sources were used: (i) case-level narrative texts from biomedical literature, from which symptom mentions were extracted using the LLM, and (ii) Orphanet disease phenotype annotations, which served as the gold-standard reference. For each disease *d*, the Orphanet phenotype profile provides a curated set of HPO identifiers categorized into *very frequent*, *frequent*, and *occasional* symptoms. We denote the gold-standard set as follows:

(14) $$G_{d}=\left\{h_{1},h_{2},\ldots ,h_{n}\right\},\;h_{i}\in \mathrm{HPO}.$$

#### 5.2.2. LLM-Assisted Symptom Extraction

For each disease, the LLM was prompted to read case descriptions and produce a structured list of symptom expressions. These raw symptom mentions were aggregated and normalized by lowercasing, whitespace normalization, and duplicate removal to obtain a final set:

(15) $$R_{d}=\left\{r_{1},r_{2},\ldots ,r_{m}\right\},$$

where each $r_{i}$ is a unique symptom expression extracted for disease *d*.

#### 5.2.3. Semantic Normalization to HPO

Because raw symptom phrases often differ lexically from their canonical HPO labels, we employed an embedding-based semantic similarity model (SentenceTransformer) to map each extracted symptom to its closest HPO concept. Each symptom phrase *r* and each Orphanet HPO label *h* were encoded as *k*-dimensional embeddings:

(16) $$e\left(r\right),e\left(h\right)\in R^{k}.$$

Cosine similarity between embeddings was computed as follows:

(17) $$\mathrm{sim}\left(r,h\right)=\frac{e(r)\cdot e(h)}{∥e(r)∥\;∥e(h)∥}.$$

A raw mention *r* was mapped to its nearest HPO concept $h^{*}$ if:

(18) $$\mathrm{sim}(r,h^{*})\geq \tau ,$$

where τ is a similarity threshold chosen empirically. The set of predicted HPO concepts for disease *d* was therefore:

(19) $$P_{d}=\left\{h^{*}|\exists r\in R_{d}:r\mapsto h^{*}\right\}.$$

#### 5.2.4. Evaluation Metrics

To quantitatively compare predicted phenotype sets $P_{d}$ with the gold-standard sets $G_{d}$, we computed standard retrieval metrics for each disease. Specifically:

(20) $$TP_{d}=|P_{d}\cap G_{d}|,\;FP_{d}=|P_{d}-G_{d}|,\;FN_{d}=\left|G_{d}-P_{d}\right|.$$

Precision, recall, F1-score, and Jaccard similarity were defined as follows:

(21) $${\mathrm{Precision}}_{d}=\frac{TP_{d}}{TP_{d}+FP_{d}},\;{\mathrm{Recall}}_{d}=\frac{TP_{d}}{TP_{d}+FN_{d}},$$

(22) $$F1_{d}=2\cdot \frac{{\mathrm{Precision}}_{d}\cdot {\mathrm{Recall}}_{d}}{{\mathrm{Precision}}_{d}+{\mathrm{Recall}}_{d}},\;{\mathrm{Jaccard}}_{d}=\frac{TP_{d}}{TP_{d}+FP_{d}+FN_{d}}.$$

To obtain global performance estimates over the 100 diseases, we computed:

- **Macro-averaged metrics**: the unweighted average of per-disease scores.
- **Micro-averaged metrics**: computed by pooling TP, FP, and FN across all diseases.
- **Mention-level accuracy**:
  (23) $$\mathrm{Accuracy}=\frac{\mathrm{Number}\;\mathrm{of}\;\mathrm{correct}\;\mathrm{symptom}\text{-}\mathrm{to}\text{-}\mathrm{HPO}\;\mathrm{mappings}}{\mathrm{Total}\;\mathrm{number}\;\mathrm{of}\;\mathrm{mapped}\;\mathrm{mentions}}.$$

The LLM extraction, semantic normalization, and evaluation were applied uniformly across all 100 diseases. All similarity computations and HPO mappings were performed using Python and the HuggingFace Transformers framework. The evaluation outputs were exported as structured JSON and CSV files to ensure reproducibility and transparent inspection of all predictions.

## 6. Results & Evaluation

In this section, we present the results of our evaluation experiments designed to evaluate the effectiveness of large language models (LLMs) in extracting disease-related phenotype sets, as well as the performance of Retrieval-Augmented Generation (RAG) for enhancing disease summaries. We report both quantitative metrics and qualitative analyses, highlighting the overall accuracy, coverage, and fidelity of the generated outputs in comparison to curated gold-standard datasets. Our results provide insights into the capabilities and current limitations of LLM-based semantic extraction and generation approaches for biomedical knowledge bases.

### 6.1. RAG Performance Evaluation

The completeness of DiseaseSummary fields before and after Retrieval-Augmented Generation (RAG), and the semantic similarity between curated and generated text are reported in this subsection.

The structural availability of nine narrative fields across all 1727 diseases is examined. Although each field is structurally present for all diseases, many entries lack textual content. Table 7 presents, for every field, the total number of diseases (*Present* = 1727) and the number of entries where the corresponding text is empty either before or after the RAG process.

As shown in Table 7, prior to RAG augmentation, several fields contained substantial amounts of missing content. For example, *Antenatal Diagnosis* had 1187 empty entries, whereas more general fields such as *Clinical Description* and *Epidemiology* had 768 and 762 empty entries, respectively. After RAG was applied, the number of empty entries decreased in all fields (e.g., from 1187 to 967 empty entries in *Antenatal Diagnosis*). This decrease demonstrates the efficacy of RAG, which leverages retrieved evidence to fill previously missing content and avoids leaving fields blank when sufficient evidence is available.

Table 8 presents the average similarity scores for each field. All fields exhibit high semantic consistency, with similarities ranging from 0.911 to 0.969. The highest alignment is observed in *Clinical Description* ($\bar{S}=0.969$), while more variable fields such as *Antenatal Diagnosis* and *Prognosis* show slightly lower but still strong similarity values ($\bar{S}=0.921$ for both). As described by Equation (13), these values summarize the overall fidelity between generated and curated content.

Taken together, Table 7 and Table 8, and Equations (11), (12) and (13) demonstrate that while RAG reduces completeness by intentionally avoiding unsupported text generation, the produced content exhibits strong semantic agreement with curated expert-written material.

### 6.2. LLM Performance Evaluation

The performance of the proposed LLM-based symptom extraction and semantic HPO normalization pipeline was evaluated across 100 randomly selected Orphanet diseases. For each disease, the predicted set of HPO concepts was compared with the corresponding gold-standard phenotype set. The evaluation metrics include precision, recall, F1-score, and Jaccard similarity, each defined mathematically in Equations (21) and (22).

#### 6.2.1. Overall Performance

Table 9 summarizes the macro- and micro-averaged performance metrics over all 100 diseases. Notably, the system achieved perfect micro-average precision (1.0) and perfect mention-level accuracy (1.0), as shown Table 10. The perfect micro-precision score is a critical finding, as it indicates that across the entire 100-disease validation set, the LLM pipeline did not generate a single false-positive (FP = 0) HPO mapping.

The macro-average precision (0.892) is lower than the micro-average. This is the expected result of a macro-average, which is penalized by the 11% of diseases in our sample for which no symptoms were confidently extracted (i.e., |P|=0), resulting in a per-disease precision score of 0.0 for those specific cases. The perfect micro-precision, however, confirms the system’s high reliability and conservative nature.

As is common for symptom extraction from highly heterogeneous, multi-source clinical text, recall was more modest, with observed values near 0.40 (macro = 0.397, micro = 0.411). This level of recall is broadly consistent with published benchmarks for rare-disease phenotyping, where natural language variability, limited case detail, and partial gold standards frequently constrain exhaustive symptom recovery. Achieving higher recall remains a well-recognized challenge in this task domain.

The macro-averaged F1 score of approximately 0.52 reflects the practical balance between perfect precision and moderate recall. Similar F1 values are routinely reported in systems tasked with extracting and normalizing symptoms from multi-source literature for rare diseases, where both description variability and phenotypic heterogeneity are pronounced, and where the reference (Orphanet) itself may be incomplete.

The Jaccard similarity index further quantifies set-level overlap, with a macro-averaged score near 0.40. This again represents the expected recovery fraction for disease-level phenotypes under stringent correctness criteria. Lastly, perfect mention-level accuracy (1.0) attests to the method’s strict avoidance of spurious or incorrect mappings—a property of paramount importance for downstream clinical and translational use.

#### 6.2.2. Per-Disease Performance

To illustrate disease-level variability, Table 10 presents a representative subset of per-disease metrics. Each disease entry reports the number of gold-standard symptoms (|G|), number of predicted symptoms (|P|), the number of true positives (TP), and the resulting evaluation metrics. In this table, the notations |G|, |P|, TP, Precision, Recall, and F1 correspond directly to the definitions given earlier. The differences across diseases reflect heterogeneity in the richness of narrative symptom descriptions.

Diseases with detailed and symptom-rich case descriptions (e.g., 51, 72, 273, 805, 636) reached high recall values (0.70–0.90) and correspondingly high F1-scores (0.75–0.90). Conversely, diseases with limited or sparse textual information (e.g., 36, 96, 782, 2940) achieved a recall of 0.00 because no extracted symptoms exceeded the similarity threshold.

Together, these results demonstrate that the proposed pipeline excels in generating high-confidence phenotype mappings, as reflected in perfect precision and mention-level accuracy while achieving moderate coverage of Orphanet disease phenotype sets. The variability in recall aligns with the degree of detail available in narrative case descriptions.

## 7. Discussion

The evaluation of our automated pipeline for mapping clinical symptom narratives to Human Phenotype Ontology (HPO) concepts highlights its notable strengths and current limitations. Chief among the strengths is the achievement of perfect micro-average precision and perfect mention-level micro-accuracy: all predicted symptom-to-HPO mappings aligned with the gold-standard Orphanet sets, demonstrating the robustness of the similarity thresholds applied in the extraction process. This outcome indicates that the system is highly conservative, effectively filtering out uncertain or spurious mappings and retaining only those with strong semantic confidence.

However, this strength in precision came at the expense of recall, which remained moderate at both macro and micro levels. The relatively low recall suggests that the pipeline, while highly selective, does not identify all relevant symptoms present in patient narratives, particularly for diseases where the available text is sparse, general, or employs less conventional language. This limitation is closely tied to the diversity of clinical text, expression variations, and heterogeneity in symptom definitions across rare disease cases. For example, synonyms, implicit references, or non-standard descriptions of symptoms may not be captured by the current similarity-based matching approach. To address this, future work could incorporate a larger vocabulary of medical synonyms, advanced NLP techniques such as contextual embeddings, and additional training data from diverse sources to improve recall without compromising precision. Iterative tuning in collaboration with clinical experts could also help refine the extraction process and enhance sensitivity to less direct symptom expressions.

As shown in the per-disease metrics, cases with richly annotated and symptom-dense text (e.g., IDs 51, 72, 273, 805, 636) achieved substantially better recall and F1-scores compared to cases with minimal or ambiguous reporting (e.g., 36, 96, 782, 2940), where the system sometimes failed to map any symptoms above the similarity threshold. This variability largely arises from the linguistic complexity, lexical diversity, and context-dependence of clinical case reports. Key phenotypic features may be described using uncommon synonyms or implicit references that are not easily captured by similarity-based matching. This trade-off between high precision and lower recall is a known challenge in automated clinical concept normalization, where maximizing confidence often means sacrificing sensitivity to less direct or non-standard expressions. Additionally, the current gold-standard (Orphanet) annotation sets may themselves be incomplete; so, some “false negatives” reflect genuine case features not previously curated rather than algorithmic misses.

Encouragingly, the pipeline’s macro F1-score remains above 0.5, reflecting meaningful overall coverage even across a heterogeneous rare disease set. These findings reinforce the critical value of detailed clinical documentation and suggest the need for continued refinement of extraction and mapping methods. Enhancing recall without compromising precision will likely require advanced natural language understanding, more comprehensive synonym handling, and iterative tuning in collaboration with clinical experts.

Another challenge stems from the heterogeneity in PubMed case report formats, which vary significantly in structure, language, and detail. This variability poses potential issues for the pipeline’s information extraction stages, particularly the LLM-based automatic reconstruction step. For example, some case reports provide detailed symptom descriptions in structured sections, while others embed clinical details within narrative prose, making extraction more challenging. To mitigate these differences, the pipeline employs flexible parsing techniques and regular-expression-based section detection. However, the impact of this heterogeneity on data quality and standardization remains a concern. Future improvements could include training the LLM on a broader range of case report formats, incorporating format-specific preprocessing steps, and leveraging metadata to identify and adapt to structural differences. Additionally, the RAG framework could be enhanced to prioritize high-quality evidence sources and flag ambiguous or incomplete entries for manual review. Addressing these challenges will further strengthen the pipeline’s robustness and its ability to handle diverse clinical text formats effectively.

## 8. Research Limitations

Despite the robust performance of our pipeline, several important limitations should be acknowledged to contextualize the results and inform future refinements. The dataset exhibits inherent selection biases owing to the predominance of case reports originating from high-resource regions and leading academic institutions, resulting in imbalanced geographic, demographic, and disease representation. Underrepresentation of certain populations, geographic areas, and ultra-rare disorders may constrain the broader generalizability and external validity of ZebraMap. Moreover, reliance on permissively licensed literature further magnifies this bias, as diseases with more open-access case reports are systematically overrepresented in the knowledge base.

A second major limitation arises from the considerable heterogeneity in clinical writing styles, narrative structure, and reporting conventions across case reports. Such variability challenges both automated section detection and semantic mapping, often reducing recall in phenotype extraction—even with advanced techniques combining rule-based and LLM-driven parsing. Nonstandard, ambiguous, or highly narrative report formats remain especially problematic for consistent information extraction. In addition, the application of large language models for field reconstruction introduces a risk of generating hallucinated or subtly inaccurate data, particularly in contexts of sparse or unclear source text. Without systematic human validation, these issues may propagate to downstream analyses. Addressing these challenges will require enhanced uncertainty quantification, automated consistency checks, and targeted human expert review, particularly for complex or ambiguous cases. Ongoing refinement of data acquisition, parsing methods, and validation protocols will be critical to further improve the data quality and scientific rigor of the knowledge base.

## 9. Conclusions and Future Directions

We introduced ZebraMap: a comprehensive, multimodal rare disease knowledge map encompassing 1727 diseases, 36,131 case reports, and 98,038 images. Our automated workflow enables standardization of case narratives and classification of associated medical images, providing a structured basis for downstream rare disease research. Through integration of retrieval-augmented generation (RAG) from the biomedical literature, we addressed knowledge gaps in disease summaries with traceable, reference-linked completions. Our baseline evaluations highlight both the reliability and the current boundaries of automated phenotype extraction in rare disease settings. Given the limitations identified, including moderate recall due to clinical text diversity, report format heterogeneity, and selection bias, future work will focus on several enhancements. We will optimize extraction and mapping to boost recall without compromising the current high precision, by expanding medical vocabularies, deploying advanced contextual NLP models, and iteratively refining methods with clinical experts. To better handle diverse report formats, we will explore format-adaptive preprocessing, broader LLM training, and metadata-guided structural alignment. Integrating genetic and genomic data is also planned, enabling robust genotype–phenotype association analyses. To address selection bias, future dataset releases will target more geographically and clinically diverse and underrepresented rare diseases. Measures such as automated uncertainty quantification, consistency checks, and targeted expert review will further strengthen data quality. Together, these improvements will ensure ZebraMap evolves into a more comprehensive, reliable, and impactful resource for rare disease research and precision medicine. 

## Figures and Tables

**Table 1:** Flow diagram of the literature search and selection process.

Stage	Number of Records
Screening	1037
Eligibility	413
Included	258
Final Included	50

**Table 2:** Summary of existing rare disease data repositories and their key limitations with respect to structured, multimodal, case-linked research.

Repository	Scope/Content	Key Limitations	Ref.
Orphanet	Rare disease ontology: disease definitions, clinical symptoms, genes, prevalence, inheritance	Lacks direct integration of medical images; not systematically linked to real-world case reports or clinical narratives	[10]
MONDO	Disease ontology unifying multiple vocabularies	Focuses on terminology unification; limited real-world patient data; lacks linkage to images or case reports	[40]
HPO (Human Phenotype Ontology)	Vocabulary of phenotypic abnormalities	Does not include case-level or multimodal (image/text) data	[40]
RD-Connect Registry & Biobank Finder	Metadata about registries and biobank samples	Provides only summary metadata; does not include raw clinical data, images, or comprehensive case-level datasets	[36]
PubMed	Biomedical research, including case reports	Not structured by disease; case reports are unlinked to standard disease IDs or images	[32]
European rare disease registries	Disease/center-specific clinical registries (demographics, features, interventions)	Siloed data; variable structure; images and unstructured narratives often unavailable; limited interoperability	[34]
MIMIC-IV	Clinical critical care EHR with narrative text, labs, images	Not rare disease-focused; very limited coverage of pediatric rare conditions; case linkage inconsistent	[49]

**Table 3:** Summary of publicly available rare disease datasets: number of cases, modalities, scope, and references.

Dataset or Registry	Scope/Focus	Number of Cases	Modalities	Ref.
Orphanet	Catalog of rare diseases, gene associations, phenotypes, epidemiology	∼6200 diseases, curated metadata	Structured text, ontologies	[2]
RD-Connect/ERDRI Registry Finder	European rare disease registries metadata	∼800 registries; ∼600,000 cases (aggregated)	Demographic, clinical, genetics (summary)	[36]
MIMIC-IV	Critical care EHR (not rare disease-specific, but includes some rare cases)	∼300,000 admissions	Text, tabular, imaging	[49]
DECIPHER	Rare disease patient variant and phenotype database	>40,000 patients	Genetics, clinical features	[53]
UDN (Undiagnosed Diseases Network)	Research cohort, undiagnosed/rare diseases	∼1800 participants (published)	Genomics, clinical notes, imaging	[54]
Face2Gene Open	Images of patients with rare genetic syndromes (for facial analysis)	>18,000 images (syndromic faces)	Imaging, clinical data	[55]
PhenomeCentral	Matchmaking and data sharing for rare and undiagnosed patients	>11,000 cases	Structured text, genotype, phenotype	[56]

**Table 4:** Key studies on multimodal knowledge maps in rare disease diagnosis, highlighting challenges.

Paper	Focus/Methodology	Data Modalities	Key Findings
[11]	Scoping review of diagnostic support systems for rare diseases	Phenotype, images, fluids	Multimodal approaches improve diagnosis, but standardization is lacking
[13]	Scoping review of multimodal machine learning in precision health	EHR, images, omics	Multimodal fusion improves predictive accuracy by ∼6.4% over unimodal
[14]	Systematic review of intermediate fusion in biomedical deep learning	Imaging, text, genetics	Intermediate fusion outperforms early/late fusion for complex tasks
[6]	Review of AI in rare diseases	Text, images, omics	AI/ML enables pattern discovery; data scarcity and bias remain issues
[16]	Scoping review of deep learning for rare disease	Images, omics, text	CNNs dominate; diagnosis is main focus; data scarcity is a challenge

**Table 5:** Summary of key claims, reasoning, and supporting papers in the literature on multimodal knowledge maps and rare disease diagnosis.

Claim	Reasoning	Papers
Multimodal knowledge bases improve rare disease diagnosis over unimodal approaches	Multiple reviews and benchmarks show higher predictive accuracy and richer knowledge representation	[6,18,41,42,55,63]
Data scarcity and lack of standardized benchmarks limit reproducibility	Most studies use private/siloed datasets; few external validations exist	[8,13,19,23,28]
LLMs enhance knowledge extraction but risk hallucination and bias	LLMs synthesize diverse data but depend on input quality and may introduce errors	[6,15,26,27]

**Table 6:** ZebraMap Dataset Properties.

Property	Count
Total number of diseases	1727
Total number of articles	36,131
Total number of text cases	69,146
Number of cases with images	45,198
Total number of images	98,038

**Table 7:** Completeness of DiseaseSummary fields before and after RAG. All fields are structurally present for all 1727 diseases. “Empty” counts indicate missing or blank textual entries.

Field	Present	Empty (Before RAG)	Empty (After RAG)
Epidemiology	1727	762	369
Clinical description	1727	768	278
Etiology	1727	773	293
Diagnostic methods	1727	842	332
Differential diagnosis	1727	882	390
Antenatal diagnosis	1727	1187	967
Genetic counseling	1727	1007	451
Management and treatment	1727	830	454
Prognosis	1727	857	400

**Table 8:** Average cosine similarity between curated and generated text across the 100-disease validation set, including overall accuracy. *N* denotes the number of valid curated–generated text pairs for each field.

Field	Average Similarity	*N*
Epidemiology	0.911	188
Clinical description	0.969	200
Etiology	0.957	188
Diagnostic methods	0.946	192
Differential diagnosis	0.950	192
Antenatal diagnosis	0.921	152
Genetic counseling	0.932	190
Management and treatment	0.951	186
Prognosis	0.921	192
**Overall Accuracy**	0.945	

**Table 9:** Macro- and micro-averaged evaluation metrics computed across 100 diseases.

Metric	Macro	Micro
Precision	0.892	1.000
Recall	0.397	0.411
F1-score	0.522	0.583
Jaccard	0.397	0.411
Mention-level Accuracy	1.000	

**Table 10:** Representative per-disease evaluation metrics. |G|= number of Orphanet gold-standard HPO terms; |P|= number of predicted HPO terms; TP= number of true positives.

Disease ID	|*G*|	|*P*|	*TP*	Precision	Recall	F1
14	51	23	23	1.00	0.451	0.622
51	63	45	45	1.00	0.714	0.833
72	70	45	45	1.00	0.643	0.783
84	106	30	30	1.00	0.283	0.441
193	64	38	38	1.00	0.594	0.745
273	91	64	64	1.00	0.703	0.826
550	84	58	58	1.00	0.690	0.817
805	49	40	40	1.00	0.816	0.899
636	74	58	58	1.00	0.784	0.879
470	70	2	2	1.00	0.029	0.056

## Data Availability

The ZebraMap dataset is publicly available at https://zenodo.org/records/17623607 (accessed on 25 May 2024). The dataset is licensed under the Creative Commons Attribution 4.0 International License (CC BY 4.0). All extracted text and images were obtained from scientific articles whose licenses permit text and data mining (TDM) and reuse for research purposes. Only articles with explicit permissive licenses (e.g., Creative Commons or publisher-granted TDM rights) were included. Each of the 36,131 case reports and 98,038 images in ZebraMap is linked to its original source publication via DOI or PubMed ID, ensuring traceability and compliance with copyright. Users of the dataset must adhere to the original article licenses when reusing any text or images.

## Supplementary Materials

The following supporting information can be downloaded at: https://www.mdpi.com/article/10.3390/diagnostics16010107/s1.

## Abbreviations

The following abbreviations are used in this manuscript:

LLM	Large Language Model
RAG	Retrieval-Augmented Generation
HPO	Human Phenotype Ontology
RD	Rare Disease
AI	Artificial Intelligence
