# Supplementary material for: ZebraMap: A Multimodal Rare Disease Knowledge Map with Automated Data Aggregation & LLM-Enriched Information Extraction Pipeline
Source: Diagnostics (Basel). 2025 Dec 29;16(1):107. doi: 10.3390/diagnostics16010107 (PMC12785374; doi:10.3390/diagnostics16010107)
Supplement: Supplementary file 1 [file diagnostics-16-00107-s001.zip › diagnostics-4023876-supplementary.pdf]

Prompt Texts Used in RAG Disease Summarization

System Instruction

```
You are a medical assistant. Use ONLY the provided abstracts for
each disease. If evidence is insufficient for a field, return
'Unknown'. Output strict JSON only.
```

Selector Few-shot Examples

```
[Selector Few-shot 1]
DISEASE: Example Disease
FIELD: Epidemiology
ABSTRACTS:
(1) Title: Aetiology focus only. Abstract: This paper explores
molecular mechanisms and pathways in vitro.
(2) Title: Population study. Abstract: We reviewed national
registries; prevalence estimated at 1/500,000–1/1,000,000 with
clusters in founder populations.
(3) Title: Case series: imaging. Abstract: MRI patterns
described; no frequency data reported.
```

Writer Few-shot Examples

```
[Writer Few-shot 1]
FIELD: Epidemiology
SELECTED ABSTRACTS (condensed):
- Registry-based studies estimate prevalence between 1/500,000
and 1/1,000,000, with founder effects in specific populations.
- Scattered case series suggest underdiagnosis outside
surveillance networks.

EXPECTED JSON:
{"G_Epidemiology":"Over 200 cases have been reported worldwide
with clustering in founder populations. Registry data suggest a
prevalence between 1/500,000 and 1/1,000,000, although estimates
vary with ascertainment and surveillance intensity. Reports
outside established networks are sporadic, implying
underrecognition rather than absence of disease. Overall, the
condition remains rare but likely underdiagnosed."}
```

Writer Prompt Template

```
DISEASE: {disease_name}
FIELD: {field}
SELECTED ABSTRACTS (each item is the full abstract text or a
condensed form; use ONLY these):
```

```
{selected_block}

TASK:
- Write ONE cohesive paragraph of 15-200 words (approx. 3-6
sentences) that answers the FIELD using ONLY the selected
abstracts.
- Do NOT include section headings or paper framing (no
'Background', 'Methods', 'We propose', 'This study',
'Objectives').
- Do NOT introduce facts not present in the selected abstracts.
- If the selected abstracts do NOT contain information for this
FIELD, return 'Unknown'.
Return exactly this JSON:
{"G_{field}":"<paragraph or Unknown>"}
```

Prompt Texts Used in Structuring Disease Cases

```
Extract comprehensive medical information from this case. Return
ONLY valid JSON.

DISEASE: {disease_name}
PROVIDED AGE: {case_age if case_age else "Unknown"}
PROVIDED GENDER: {case_gender if case_gender else "Unknown"}

CASE TEXT:
{case_text}
{img_info}

Return this exact JSON structure (fill all fields based on the
text):
{
  "Department": "<select ONE from: Neurology, Pediatrics,
Gastroenterology, General Medicine, Cardiology, Dermatology,
Oncology, Orthopedics, Pulmonology, Endocrinology, Psychiatry,
Nephrology, Rheumatology, Infectious Disease, Obstetrics and
Gynecology, Urology, Ophthalmology, Otolaryngology>",
  "Demographics": {
    "Age": <integer, floor value, minimum 1>,
    "Gender": "<Male/Female/Unknown>",
    "ByBirth": "<Yes/No/Unknown>",
    "Genetic": "<Yes/No/Unknown>",
    "ResponsibleGene": "<gene name or empty string>"
  },
  "Diagnosis": {
    "DiseaseName": "{disease_name}",
    "Symptoms": ["<symptom1>", "<symptom2>"],
```

```
    "DiagnosisMethods": ["<method1>", "<method2>"],
    "Treatment": "<treatment description or empty>",
    "TreatmentWorked": "<Yes/No/Partial/Unknown or empty>"
  },
  "DifferentialDiagnosis": ["<disease1>", "<disease2>"],
  "Images": [
    {"FileID": "<id>", "Type": "<main category>", "SubType":
"<specific type>"}
  ]
}
```

CRITICAL RULES:
- Use PROVIDED AGE/GENDER if available, otherwise extract from text
- Age must be integer (floor/round down), minimum 1
- Department must be ONE from the exact list provided
- Image Type/SubType must match from: Trauma and Emergency Imaging, Diagnostic Imaging, Endoscopic and Minimally Invasive Imaging, Ophthalmology Imaging, Dental Imaging, Histopathology and Cytology Imaging, Cardiology Imaging, Radiation Therapy Imaging...
- Empty arrays [] or empty strings "" are acceptable when info is missing
- NO hallucination - only extract what's explicitly stated
